# Rutin attenuates complete Freund’s adjuvant-induced inflammatory pain in rats

**DOI:** 10.22038/ijbms.2024.81572.17655

**Published:** 2025

**Authors:** Fatemeh Forouzanfar, Ali Mohammad Pourbagher-Shahri, Amir Mahmoud Ahmadzadeh

**Affiliations:** 1 Medical Toxicology Research Center, Faculty of Medicine, Mashhad University of Medical Sciences, Mashhad, Iran; 2 Department of Radiology, School of Medicine, Mashhad University of Medical Sciences, Mashhad, Iran

**Keywords:** Anti-inflammatory, Anti-oxidant, Pain, Rheumatoid arthritis, Rutin

## Abstract

**Objective(s):**

Rutin is a bioflavonoid compound renowned for its anti-oxidative, anti-inflammatory, and antinociceptive properties. The present study aims to assess its therapeutic efficacy on complete Freund’s adjuvant (CFA)induced inflammatory pain.

**Materials and Methods:**

Arthritis was induced in Wistar rats via subcutaneous administration of CFA into the right hind paw. Rutin (15 and 30 mg/kg) and indomethacin (5 mg/kg, orally) were given once daily for three weeks. Parameters observed included alterations in paw swelling perimeter, arthritis scores, and body weight. Additionally, antinociceptive activity was measured through thermal hyperalgesia and cold allodynia responses. The Tumor necrosis factor-alpha (TNF-α) level in the serum was measured. Malondialdehyde (MDA), thiol levels, catalase, and superoxide dismutase (SOD) activities were also evaluated as serum oxidative stress markers.

**Results:**

Rutin and indomethacin significantly suppressed alterations in paw edema, pain responses, and arthritis scores and reduced the loss of body weight in contrast to disease-control rats. Furthermore, in contrast to disease control rats, rutin and indomethacin treatment exhibited an anti-inflammatory effect through a marked reduction in TNF-α levels in the serum. Rutin and indomethacin demonstrated a significant increase in catalase and SOD activities, a total thiol level, and a decrease in MDA level compared to the disease-control rats.

**Conclusion:**

These results suggest that rutin’s antiarthritic effect is mediated by its antinociceptive, anti-oxidant, and anti-inflammatory properties.

## Introduction

Rheumatoid arthritis (RA) is a multi-system disease with an autoimmune inflammatory nature. It primarily affects multiple joints. If not adequately managed, RA can damage synovial, cartilage, and bone structures and cause extrajoint symptoms (1-3). The exact pathophysiological basis of the disease remains elusive. However, the recruitment and overactivity of immune cells at the site of inflammation are thought to play pivotal roles in disease progression. Activated macrophages at the site of inflammation release pro-inflammatory cytokines, exacerbating the disease (4). RA is characterized by an imbalance between pro- and anti-inflammatory cytokines, favoring the former, inducing inflammation and joint damage (5, 6). Oxidative stress has been implicated in RA development, causing damage to DNA, lipids, and proteins and triggering synovial inflammation (7, 8).

The primary treatment for RA involves medications, which are typically categorized into three groups: disease-modifying antirheumatic drugs, non-steroidal anti-inflammatory drugs (NSAIDs), and biological agents. However, these medications often entail noticeable side effects (9). Consequently, alternative safe therapeutic methods warrant investigation (10).

It is not hyperbole to state that the usage of herbal remedies for medicinal purposes dates back to the dawn of humankind. They have been used for centuries to treat several illnesses. For hundreds of years, generations of practicing physicians from the traditional medical system have synthesized herbal medicines based on their therapeutic experiences (11-13). These days, researchers are very interested in medicinal substances generated from plants because the pharmaceuticals already on the market are either very expensive or have specific adverse effects. Nature has given us an immense abundance of medicinal plants found worldwide that can be used to prevent and treat several illnesses (11-13). 

Rutin (3,3′,4′,5,7-pentahydroxyflavone-3-rhamnoglucoside) is a flavonoid of the flavonol class present in various plants like apples, buckwheat, tea, and passion flowers. It exhibits numerous medicinal properties, including antimicrobial, antifungal, vasoprotective, anti-inflammatory, anti-oxidant, neuroprotective, and cardioprotective effects (14-18). Furthermore, rutin shows promise against chronic diseases such as diabetes, cancer, and hypertension (19). Previous studies showed rutin’s antinociceptive effect in different animal pain models;

In one study, the administration of rutin reversed chemical hyperalgesia duringboth phases of the formalin test in diabetic rats (20). Another study revealed that rutin ameliorated inflammation pain induced by high concentrations of ATP (21). Rutin, isolated from *Polygala paniculata*, produced dose-related inhibition of glutamate-induced pain in mice (22). Given the existing literature on rutin’s anti-oxidant, anti-inflammatory, and antinociceptive effects, we sought to evaluate its potential therapeutic impact in a rat model of complete Freund’s adjuvant (CFA)-induced RA. 

## Materials and Methods

### Animals

This study used adult male rats (180–220 g). The rats were provided from the animal Unit at the Mashhad University of Medical Sciences (MUMS), Mashhad, Iran. The rats were maintained in standard cages in environmentally controlled rooms with 22–25 °C temperature and 12-hour light-dark cycles, and sufficient water and food were given. All animal experiments were carried out under the MUMS Ethics Committee Acts (ethical code: IR.MUMS.MEDICAL.REC.1400.801).

### Induction of experimental arthritis

Arthritis was induced by a single subcutaneous injection of 100 μl of CFA (Sigma-Aldrich (St. Louis, MO, USA)) into the right hind footpad of the rats on day 0.

### Animals grouping

The rats were assigned at random into five groups, each with eight rats:

Group 1: Control rats received a 100 μl saline injection in the right hind paw.

Group 2: CFA- Rats received a 100 μl CFA injection in the right hind paw.

Group 3: Indomethacin- Rats were treated with indomethacin (5 mg/kg).

Group 4: Low-dose rutin- Rats were treated with rutin (15 mg/kg).

Group 5: High-dose rutin- Rats were treated with rutin (30 mg/kg).

Based on prior studies, the dosage of rutin was selected (20, 23). 

Treatments were administered daily for 21 consecutive days of arthritis induction.

Rutin and indomethacin were dissolved in saline containing 1% DMSO just before oral administration. Paw swelling perimeter, arthritis score, and nociceptive behavioral tests were assessed on days -1, 3, 6, 9, 12, 15, 18, and 21. 

### Measurement of paw volume and arthritis score

Paw thickness was measured using a caliper. Disease severity was scored on a scale from 0 to 4 for each limb, with criteria as follows: “0 = no change, 1 = mild erythema or swelling of the digits, 2 = moderate swelling and erythema, 3 = severe swelling and erythema involving the ankle, and 4 = ankylosis and inability to bend the ankle (24).

### Thermal hyperalgesia

A hot plate (ALFA D500) set at 55 °C was used to measure thermal hyperalgesia (25). The pain threshold was considered when the rat licked or withdrew its foot. To prevent damage to the rats’ paw tissues during the experiment, a maximum cut-off time of 20 sec was applied (25).

### Cold allodynia

Rats were acclimated in Plexiglas chambers for 15 min and then placed on a metal mesh floor to examine the plantar surface of their right foot. Acetone was applied to the plantar surface of the right foot using a 10-mL syringe. Acetone evaporation elicits a stimulus that may induce reactions such as shaking or licking the injured paw. If any of these signs were observed, a positive response was recorded. Five consecutive trials were conducted, with a five-minute interval between each. The percentage calculation used the following formula: (Number of trials with response) × 100 / (Number of total trials) (26).

### Assessment of inflammation and oxidative stress

At the end of the treatment period, animals were deeply anesthetized by ketamine (100 mg/kg, IP) and xylazine (10 mg/kg, IP), and blood samples were collected. The blood samples were collected and accumulated from the abdominal aorta. After blood serums were separated by centrifuge, the isolated serum was maintained at −80 °C until use. Serum tumor necrosis α (TNF-α) level was measured using a commercial enzyme-linked immunosorbent assay (ELISA) kit (Karmania Pars Gene Company, Kerman, Iran). Serum levels of malondialdehyde (MDA) and total thiol, along with catalase and superoxide dismutase (SOD) activities, were determined using previously described methods (27).

### Data statistical analysis

Comparisons among groups for behavioral tests were made by two-way analysis of variance (ANOVA) followed by Bonferroni’s post hoc test. For biochemical results, one-way ANOVA was performed, followed by Tukey’s post hoc test. Statistical analyses were carried out using Graph Pad Prism (Version 6.0) and showed the mean ± standard error of the mean (SEM). The statistical significance threshold was a *P*<0.05.

## Results

### Effect of rutin on animal’s body weight, arthritis score, and paw volume

**Figure 1:**
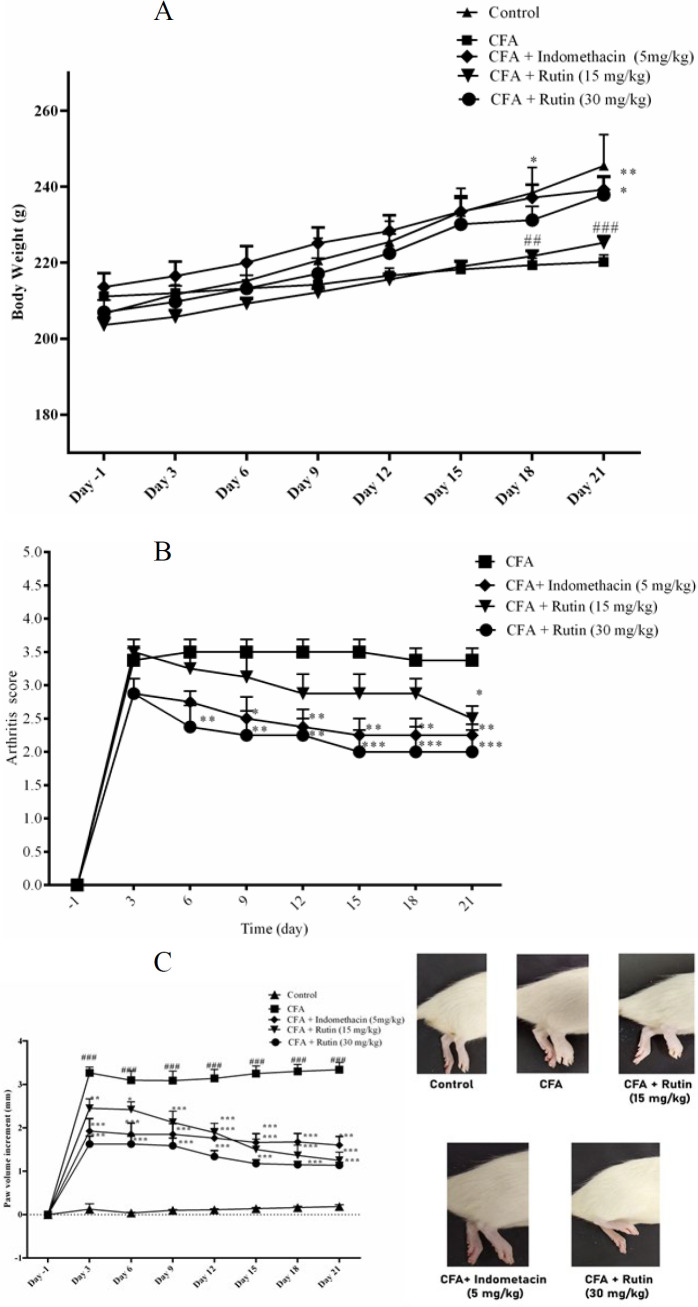
Effects of rutin (15 and 30 mg/kg) and indomethacin on (A) body weight, (B) Arthritis Score, and (C) paw volume changes in rats with CFA-induced inflammatory pain

Throughout the study, control rats exhibited consistent body weight gain. However, vehicle-treated CFA rats significantly decreased body weight on days 18 and 21 compared to the control rats (*P*<0.01, *P*<0.001, respectively). Indomethacin-treated rats exhibited a significantly higher body weight than the vehicle-treated CFA group on days 18 and 21 (*P*<0.05 and *P*<0.01, respectively). Moreover, administration of rutin at a high dose (30 mg/kg) significantly increased the body weight on day 21 versus the vehicle-treated CFA group (*P*<0.05) (Figure 1A).

Figure 1B revealed that the initial arthritis score was 0 for all groups on day 0. Indomethacin treatment significantly diminished the arthritis index versus the vehicle-treated CFA on day 9 (*P*<0.05) and consistently from day 12 through day 21 (*P*<0.01). 

Rutin-treated rats (15 mg/kg) displayed a lower arthritis score versus the vehicle-treated CFA on day 21 (*P*<0.05). Rutin-treated rats (30 mg/kg) demonstrated lower arthritis scores compared to the vehicle-treated CFA from day 6 through day 21 (days 6, 9, 12: *P*<0.01; Days 15, 18, and 21: *P*<0.001, respectively). 

As illustrated in Figure 1C, the vehicle-treated CFA group demonstrated a significant increase in paw volume from day 3 through day 21 compared with the control group (*P*<0.001). Rutin-treated rats (15 mg/kg) exhibited a significantly lower paw volume versus the vehicle-treated CFA group from day 3 through day 21 (day 3: *P*<0.01; day 6: *P*<0.05; and day 9 to 21: *P*<0.001, respectively). Rutin-treated rats (30 mg/kg) and indomethacin-treated rats (5 mg/kg) led to a significant reduction in paw volume compared to the vehicle-treated CFA group from day 3 through day 21 (*P*<0.001).

### Effect of rutin on thermal hyperalgesia (Hot plate)

**Figure 2:**
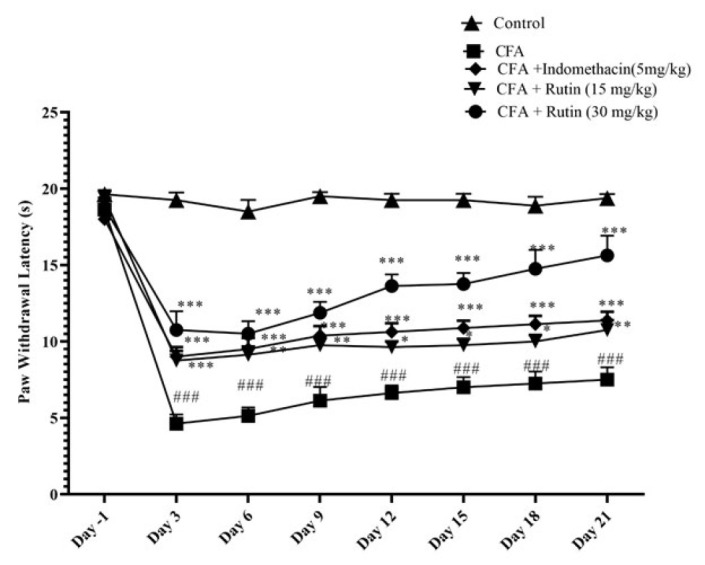
Effects of rutin (15 and 30 mg/kg) and indomethacin on thermal hyperalgesia s in rats with CFAinduced

Compared with the control group, the vehicle-treated CFA group demonstrated an obvious decline in the paw withdrawal latency from day 3 through day 21 (*P*<0.001). Conversely, rats treated with either a high dose of rutin (30 mg/kg) or indomethacin (5 mg/kg) manifested an increase in paw withdrawal latency from day 3 up until day 21 versus the CFA-administered rats (*P*<0.001). Likewise, Rutin-treated rats (15 mg/kg) also showed an elevated paw withdrawal latency from day 3 through day 21 in comparison to the CFA-administered group (Day 3: *P*<0.001; day 6: *P*<0.01; day 9: *P*<0.01; days 12 to 18: *P*<0.05; day 21: *P*<0.01, respectively) (Figure 2).

### Effect of rutin on cold allodynia

**Figure 3:**
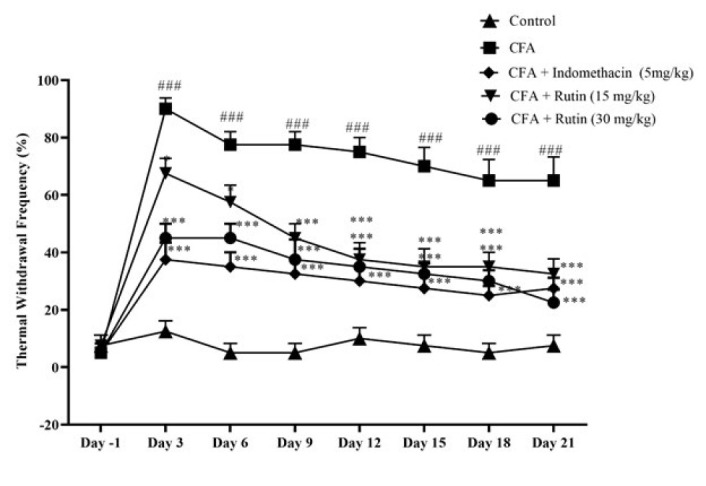
Effect of rutin (15 and 30 mg/kg) and indomethacin on cold allodynia in rats with CFAinduced inflammatory pain

CFA rats exhibited a significant increase in thermal withdrawal frequency in the cold allodynia test from day 3 to day 21 versus the control group (*P*<0.001). Rats treated with a high dose of rutin (30 mg/kg) or indomethacin (5 mg/kg) demonstrated a markedly lower thermal withdrawal frequency from day 3 to day 21 versus the CFA-administered rats (*P*<0.001). Similarly, rutin-treated rats (15 mg/kg) showed a decreased thermal withdrawal frequency from day 3 through day 18 compared to rats administered solely with CFA (Days 3 and 6: *P*<0.05, days 9 to 18: *P*<0.001, respectively) (Figure 3).

### Effect of rutin on the serum level of TNF-α

**Figure 4:**
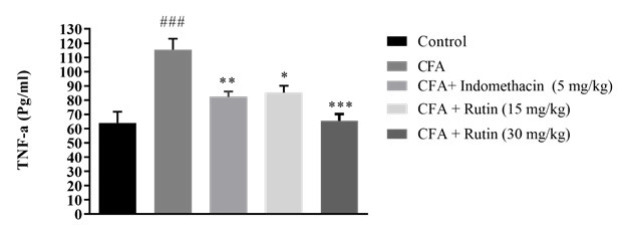
Effects of rutin (15 and 30 mg/kg) and indomethacin (5 mg/kg) on total serum TNF-α level in rats with CFA-induced inflammatory pain

TNF-α levels in the vehicle-treated CFA group were significantly higher versus the control group (P<0.001, Figure 4). Indomethacin-treated rats exhibited significantly lower TNF-α levels than CFA-induced rats (P<0.01). Further, the serum TNF-α levels in rats treated with rutin (both 15 and 30 mg/kg doses) were significantly lower compared to those in the CFA-administered rats (P<0.05 and P< 0.001, respectively).

### Effect of rutin on the serum oxidative stress markers

**Figure 5:**
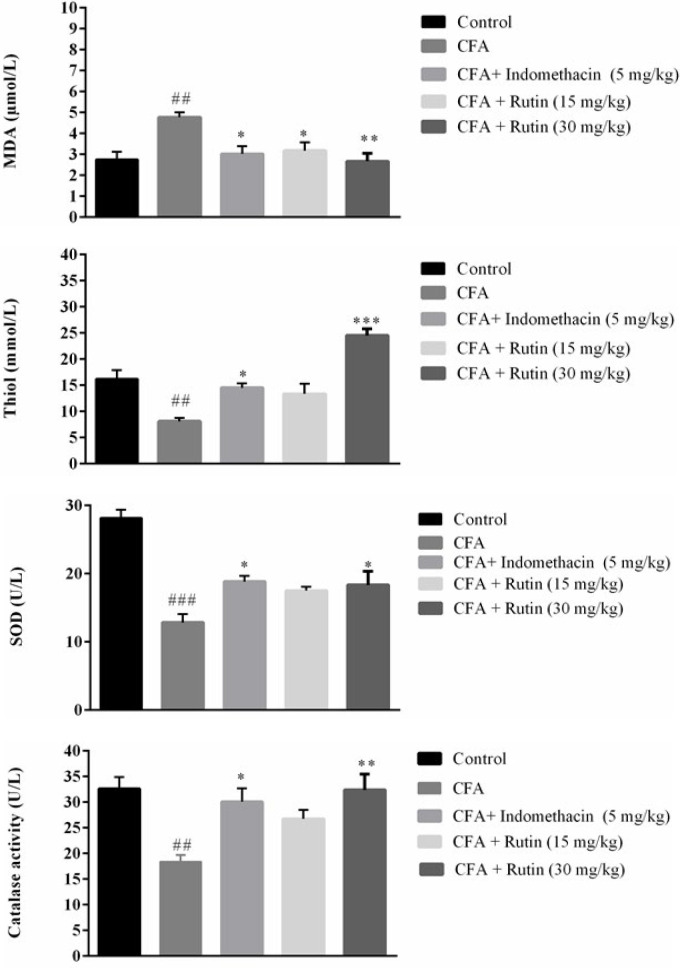
Effects of rutin (15 and 30 mg/kg) and indomethacin (5 mg/kg) on total serum (A) MDA, (B) Thiol levels, (C) SOD, (D) Catalase activities in rats with CFA-induced inflammatory pain

Serum MDA level was significantly higher in the vehicle-treated CFA group versus the control group (P<0.01). Indomethacin-treated rats exhibited a significantly lower MDA level than CFA-induced rats (*P*<0.05). Likewise, significantly lower total serum MDA levels were found in the rats treated with either dose of rutin (15 mg/kg and 30 mg/kg) versus the CFA group (*P*<0.05 and *P*<0.01, respectively) (Figure 5A).

A significant diminution in the serum level of total thiol was found in the vehicle-treated CFA group versus the control rats (P<0.01). As depicted in Figure 5B, the rats treated with a high dose of rutin or indomethacin displayed significantly higher total thiol levels than those of the CFA-administered rats (*P*<0.001 and *P*<0.05, respectively). SOD activity was significantly diminished in the rats that received only CFA versus the control group (*P*<0.001). The rats treated with either a high dose of rutin or indomethacin demonstrated significantly elevated levels of SOD versus the CFA group (both *P*<0.05) (Figure 5C). Finally, a significant decline in catalase activity was found in the CFA group compared to the control group (*P*<0.01). Treatment with either a high dose of rutin or indomethacin significantly enhanced catalase activity in contrast to the CFA-administered rats (*P*<0.01 and *P*<0.05, respectively) (Figure 5D).

## Discussion

In this study, we intended to elucidate the impact of rutin on CFA-induced RA in a rat model. The results demonstrated that both rutin and indomethacin impeded the progression of RA, evidenced by significant reductions in inflammation, oxidative stress, arthritis scores, pain responses, and paw edema.

The CFA-induced arthritis animal model is widely recognized for investigating arthritis pathogenesis and assessing the efficacy of antiarthritic drugs. Numerous inflammatory reactions have been associated with CFA administration, including cutaneous inflammation characterized by redness and swelling at the injection site on the rat’s footpad (28, 29). A potential early stage of RA involves activating the innate immune system, which initiates a T-cell response likely targeting citrullinated proteins. Upon entry into the synovial layer, T-cells can activate other inflammatory cells and synovial fibroblasts through direct contact and cytokine stimulation, including TNF-α, interferon-γ, and interleukin (IL)-17. Activation of these cells further leads to the production of inflammatory cytokines (30). 

Potent pro-inflammatory cytokine TNF-α, often referred to as cachectin, is critical to the immune system during cell proliferation, apoptosis, and inflammation. Various cells can produce TNF-α, although macrophages and T-cells are believed to be the primary producers. These cells include mast cells, B cells, fibroblasts, NK cells, neutrophils, smooth muscle cells, endothelial cells, cardiomyocytes, osteoclasts, osteoblasts, microglial cells, adipocytes, astrocytes, dendritic cells, glomerular mesangial cells, adrenocortical cells, and keratinocytes (31). 

Dysregulation of cytokines, such as TNF-α and IL-1β, leads to synovial hyperplasia and gradual degeneration of joints, in addition to triggering inflammation. TNF-α and IL-1β play pivotal roles in the pathogenesis of RA by enhancing immune responses, inducing the production of other inflammatory cytokines, promoting osteoclast differentiation, and accelerating joint destruction, thereby initiating a vicious cycle (32). Accordingly, TNF-α was selected in the present study to assess inflammation, and its level was revealed to be higher in the serum of RA rats, indicating inflammation induced by CFA. Both rutin and indomethacin significantly suppressed inflammation in the CFA model.

One study previously reported the anti-inflammatory effect of rutin, which showed that rutin reduced neutrophil recruitment, joint edema, mechanical hypernociception, and articular TNF-α synthesis in murine zymosan-induced arthritis (33). 

When the body’s anti-oxidant defense systems are out of balance with free radical molecules like reactive oxygen species (ROS) or reactive nitrogen species (RNS), oxidative stress is induced(34, 35). Overly high concentrations of free radicals harm vital cell components such as proteins, lipids, and nucleic acids, which ultimately results in damage and apoptosis. The anti-oxidant defense system prevents harm by scavenging excess free radicals under normal circumstances. The upregulation of ROS may be the underlying mechanism for painful conditions(36). Oxidative stress is also involved in the pathogenesis of RA (25). There are reports of altered anti-oxidant systems and elevated lipid peroxidation levels in serum and synovial fluid in RA. Indirect evidence supporting the role of ROS in ligament deterioration includes the presence of nitrous type II collagen peptide, modified low-density lipoprotein, oxidized IgG, and cartilage peroxidation products in the blood and urine of patients with RA (30). Pro-inflammatory cytokines are produced in response to ROS generation. Moreover, the inflammation process, which involves host immune cells producing significant levels of ROS through the NADPH oxidase enzyme pathway, also contributes to oxidative stress (37). 

Lipid peroxidation occurs when oxidants, such as free radicals, attack lipids that contain one or more carbon-carbon double bonds, mainly polyunsaturated fatty acids. This process produces hydroperoxides and lipid peroxyl radicals(38). The major mutagenic byproduct of lipid peroxidation seems to be MDA(38). Our findings showed that rutin reduced MDA levels.

It has been demonstrated that SOD can produce hydrogen peroxide and oxygen from oxygen-free radicals generated by xanthine oxidase. The primary anti-oxidant defense mechanism within cells against free radicals is thought to be SOD. SOD has been connected to preventing DNA damage in susceptible situations and safeguarding cellular structure. *In vitro* and *in vivo* have demonstrated that an exogenous SOD supplement may shield cells from chemically induced cell lysis and delay cellular death (39).

Catalase is a crucial enzyme that neutralizes and breaks down hydrogen peroxide, keeping the molecule at an optimal level in the cell—a requirement for cellular signaling processes. The significance of the enzyme can be determined by the fact that it is involved directly or indirectly in many illnesses and infections (40).

According to earlier research, oxidative stress and RA are linked to higher serum levels of lipid peroxidation, lower levels of glutathione, and lower activities of SOD and catalase (41, 42). Our findings corroborated the data mentioned above, showing that rutin restored anti-oxidant markers, including SOD, catalase, and total thiol.

One study showed that treatment with rutin alleviated hyperalgesia and improved motor coordination in diabetic rats. Streptozotocin dramatically elevated thiobarbituric acid reactive substances and lowered glutathione levels in the sciatic nerve, although rutin therapy protected against these changes considerably. Catalase, SOD, glutathione peroxidase, glutathione-S-transferase, and glutathione-reductase activities were markedly diminished in diabetic rats. Rutin therapy dramatically improved anti-oxidant protection (43).

CFA-induced RA in rats mimics human RA, including polyarticular inflammation and heat and mechanical hypersensitivity (44, 45). Additionally, RA-related joint inflammation leads to central sensitization, which is characterized by the emergence of allodynia and hyperalgesia (45). 

CFA induces chronic inflammatory pain in experimental rats by upregulating IL-1β, IL-6, and TNF-α, heightening pain receptors’ sensitivity and lowering pain thresholds (45, 46). We found significantly reduced thermal hyperalgesia and cold allodynia in the rats treated with either doses of rutin or indomethacin. In a similar study, administering a rutin-containing medicinal plant or celecoxib significantly attenuated thermal and mechanical hyperalgesia (46). 

Rutin has been shown to have antinociceptive effects in numerous animal models of pain;

Both systemic and intra-ventrolateral periaqueductal grey matter administration of rutin have been demonstrated to produce an antinociceptive effect, partially mediated by an opioidergic mechanism (47). Rutin has been found to mitigate various aspects of allodynia and spontaneous pain. It blocked the activation of mitogen-activated protein kinases and reduced inflammatory or pain-stimulating mediators in the dorsal root ganglion of neuropathic rats. In addition, rutin treatment reduced neuroinflammation and microglial activation in the spinal cord of neuropathic rats (48). Another study showed that rutin and naproxen have synergistic analgesic interaction in the acetic acid-induced writhing test in mice (49). Rutin treatment attenuated punctate allodynia in neuropathic rats. Rutin also reduced dynamic allodynia and the spontaneous pain behavior score. Furthermore, induction of neuropathic significantly increased the expression of pronociceptive genes such as CX3C chemokine receptor 1, C-C chemokine receptor type 2, and matrix metallopeptidase-9 in the dorsal root ganglion. These pronociceptive genes were all inhibited by rutin administration (48).

Evaluating the antiarthritic potential of various drugs typically involves assessing the arthritis score and paw swelling (50, 51). This research examined rutin using these indices, and it was found to significantly diminish paw thickness and arthritis score.

Indomethacin is an NSAID drug with antipyretic, analgesic, and anti-inflammatory properties. Its mechanism of action hinges on inhibiting cyclooxygenase activity, leading to a subsequent decrease in prostaglandin synthesis. Despite its notable effectiveness in alleviating symptoms of certain arthritic conditions, it has been demonstrated that indomethacin does not alter the disease’s progression (52). In the present study, indomethacin demonstrated antinociceptive effects and reduced paw edema. Moreover, indomethacin lowered TNF-α levels and mitigated oxidative stress.

The current study has some limitations. First, only changes in the serum and pain-related behaviors were observed in the current investigation, and changes in pain-modulated pathways were not examined. Second, our follow-up duration was relatively short, and therefore, our results may not be indicative of long-term effects. Hence, we suggest future studies consider longer follow-up periods.

The data are expressed as mean ± SEM, (n = 8) ***P<0.001, **P<0.01, and *P<0.05 vs CFA group. ### *P*<0.001 vs control group. CFA: Complete Freund’s adjuvant.

inflammatory painPaw withdrawal latencies in the hot plate test are measured in seconds. The data are expressed as mean ± SEM. (n = 8). ***P<0.001, **P<0.01, and *P<0.05 vs CFA group. ### *P*<0.001 vs control group. CFA: Complete Freund’s adjuvant.

The data are expressed as mean ± SEM. (n = 8). ***P<0.001, and *P<0.05 vs CFA group. ### *P*<0.001 vs control group. CFA: Complete Freund’s adjuvant.

The data are expressed as mean ± SEM, (n = 6) ***P<0.001, **P<0.01, and *P<0.05 vs CFA group. ### *P*<0.001 vs control group. CFA: Complete Freund’s adjuvant.

The data are expressed as mean ± SEM, (n = 6) ***P<0.001, **P<0.01, and *P<0.05 vs CFA group. ### *P*<0.001, and ## *P*<0.01 vs control group. CFA: Complete Freund’s adjuvant.

## Conclusion

Administration of rutin in CFA-treated rats ameliorated oxidative stress, attenuated TNF-α, and improved the results of thermal hyperalgesia and cold allodynia tests. Hence, rutin could alleviate CFA-induced arthritis by exerting anti-oxidant, anti-inflammatory, and antinociceptive properties. Future research may reveal other potential mechanisms of rutin’s action in RA. Furthermore, clinical trials are needed to confirm its efficacy in humans.

## Data Availability

The datasets used for analysis in this study are available upon request from the corresponding author.

## Declaration

We have not used any AI tools or technologies to prepare this manuscript.
